# Supplementary material for: Deletion of wfs1 Impairs Oligodendrocyte Precursor Cells Dorsal Distribution and Myelination Through the wfs1-hmgcs1 Axis in Zebrafish
Source: Biology (Basel). 2026 Aug 16;15(16):1402. doi: 10.3390/biology15161402 (PMC13510151; doi:10.3390/biology15161402)
Supplement: Supplementary file 1 [file biology-15-01402-s001.zip › File S1-Transcriptome sequencing data and differentially expressed genes (DEGs) in wfs1-- zebrafish at 5 dpf/2.KEGG/wfs1KOvsWT/UP/src/dre04310.html]

dre04310


- dre:100006675

- Up regulated genes

100006675(1.63301233543285)

- dre:565241

- Up regulated genes

565241(1.32977867526604)

- dre:58072

- Up regulated genes

58072\_1(2.20804115837263)

- dre:321070

- Up regulated genes

101882911(3.50047007681359)

- dre:436718

- Up regulated genes

436718(1.34229427553561)

- dre:58072

- Up regulated genes

58072\_1(2.20804115837263)

- dre:564241

- Up regulated genes

564241(1.44141542620362)

- dre:678649

- Up regulated genes

678649\_1(5.62990189430434)

- dre:566841

- Up regulated genes

566841(1.91411798034227)

- dre:58072

- Up regulated genes

58072\_1(2.20804115837263)

- dre:556087

- Up regulated genes

556087(1.25789886083115)

- dre:100000500

- Up regulated genes

100000500\_1(1.68235667540684)

- dre:103909653

- Up regulated genes

103909653(1.03835456243127)

Close
